# Safety, Tolerability, and Immunogenicity of the TANCoV-1.3.20 SARS-CoV-2 Vaccine Among Healthy Participants in Tanzania: Protocol for a Multisite, Phase 1/2a, Double-Blinded Randomized Controlled Trial

**DOI:** 10.2196/81323

**Published:** 2026-05-11

**Authors:** George Peter Judicate, Marco Missanga, Mbazi Senkoro, Emmanuel Kapesa, Nhamo Chiwerengo, Redemta Ferdinand, Gibson Kagaruki, Godfather Kimaro, Esther Ngadaya, Sokoine Lesikari Kivuyo, Deus Buma, Lwitiho Sudi, Abisai Kisinda, Lucas Maganga, Doreen Pamba, Joel Mwakisisile, Willyhelmina Olomi, Nyanda Ntinginya Elias, Said Aboud, Philemon Wambura, Sayoki Mfinanga

**Affiliations:** 1 Research Department, National Institute for Medical Research, Muhimbili Center, PO Box 3436, Dar es Salaam, United Republic of Tanzania, 255 222121400; 2 Department of Microbiology and Immunology, Muhimbili University of Health and Allied Sciences, Dar es Salaam, United Republic of Tanzania; 3 Research Department, National Institute for Medical Research, Mbeya, United Republic of Tanzania; 4 Data Management, National Institute for Medical Research, Mbeya, United Republic of Tanzania; 5 Department of Pharmacy, Muhimbili National Hospital, Dar es Salaam, United Republic of Tanzania; 6 Research Department, National Institute for Medical Research, Headquarters, Dar es Salaam, United Republic of Tanzania; 7 Department of Microbiology, Sokoine University of Agriculture, Morogoro, United Republic of Tanzania

**Keywords:** COVID-19, COVID-19 vaccines, SARS-CoV-2, avian coronavirus, ACoV, nasal vaccine, intranasal vaccine, Sinopharm, acceptability

## Abstract

**Background:**

The COVID-19 pandemic has caused devastating morbidity and mortality globally, and poses an unprecedented threat to economic growth. The global rollout of vaccines has been met with socioeconomic disparities, impeding the global effort in infection prevention and severity reduction. The development and evaluation of candidate vaccines against COVID-19 that overcome logistical, social, and economic challenges are highly needed. Here, a trial protocol is presented to assess the safety, tolerability, and immunogenicity of the TANCoV-1.3.20 SARS-CoV-2 vaccine among healthy participants who were SARS-CoV-2 negative in Tanzania.

**Objective:**

The primary objective of this study was to evaluate the safety and tolerability of the TANCoV-1.3.20 nasal SARS-CoV-2 vaccine in healthy adult participants in Tanzania. The secondary objectives included the assessment of vaccine-induced humoral and cellular immune responses, and preliminary evaluation of dose-related immunogenicity.

**Methods:**

TANCoV-1 is a phase 1/2a double-blinded, randomized controlled trial conducted in the Dar es Salaam and Mbeya regions of Tanzania. A total of 150 healthy participants were planned to be recruited and randomized at a 1:1:1 ratio to receive a TANCoV-1.3.20 vaccination dose of 100 µL with or without a booster, or 200 µL without a booster. Each dose group was planned to contain an intervention arm and a control arm in a 1:1 ratio. In general, 75 participants were planned to be assigned to the experimental arm, and another 75 participants were planned to be assigned to the standard arm. Participant recruitment was expected to take 6 months, with follow-up for 6 months post vaccination. The trial has two primary end points: (1) safety, as ascertained by the incidence of adverse events, and (2) immunogenicity, which involves local, humoral, and cellular immune responses. The trial enrolled healthy individuals aged between 18 and 45 years, who provided written informed consent and met all the inclusion criteria per the protocol. The data will be analyzed using Stata (version 14; StataCorp LLC). Findings will be reported according to the CONSORT (Consolidated Standards of Reporting Trials) guidelines.

**Results:**

This paper describes the study protocol. Recruitment was completed in February 2024, following regulatory and ethical approvals. Safety and immunogenicity data will be analyzed after the completion of participants’ follow-ups. Laboratory testing is ongoing, and data cleaning and statistical analyses are underway. Safety and immunogenicity outcomes will be analyzed after all follow-up visits and laboratory assays have been completed.

**Conclusions:**

This randomized clinical trial in an African context will provide valuable data that can be used to ensure the future availability of safe, cost-effective, and environmentally accustomed effective vaccines. Furthermore, the findings of this trial will help increase public awareness and acceptance of COVID-19 vaccines, contributing to efforts to combat the COVID-19 pandemic.

## Introduction

### COVID-19 and Its Origin

COVID-19 is a respiratory illness caused by SARS-CoV-2, a virus that was first reported in Wuhan, China, in 2019 [1]. This disease was declared a pandemic by the World Health Organization (WHO) in 2020 because of the large number of cases reported globally [2,3]. As of July 9, 2023, there were more than 767 million confirmed cases and more than 6.9 million deaths reported globally, with the majority of cases coming from Europe, the United States, and the Western Pacific. However, Africa has reported only 1% of cases and 3% of deaths globally, although this may be due to concerns about inadequate and unreliable testing facilities. According to the WHO COVID-19 dashboard, as of July 5, 2023, there were 43,078 confirmed COVID-19 cases and 846 deaths in Tanzania [4-6].

SARS-CoV-2 is a virus that belongs to the beta-genera of coronaviruses, which include other human coronaviruses, such as SARS-CoV and Middle East respiratory syndrome coronavirus (MERS-CoV) [2]. A phylogenetic analysis of several SARS-CoV-2 isolates revealed that they are genetically closely related to coronaviruses that have been isolated from bat populations, indicating that SARS-CoV-2 is a zoonotic virus that has made its way into the human population. However, as of 2020, zoonotic linkages have not been established [7]. Coronaviruses are a large family of viruses that cause a range of illnesses, ranging from mild to severe, including the common cold and more serious diseases, such as SARS-CoV, MERS-CoV, and SARS-CoV-2, which have had alarming impacts on humans [8-10].

The SARS-CoV-2 virus has evolved and changed due to genetic selection caused by mutations and recombination [11]. A high rate of mutation in SARS-CoV-2 is associated with the rapid emergence of new viral variants. For example, the alpha variant first appeared in Great Britain in November 2020, followed by the delta variant, which dominated the summer of 2021, and at the end of 2021, the omicron variant emerged and dominated the world [12].

The recommended preventive measures for SARS-CoV-2 are threefold. First, infection control should be practiced in areas where community transmission is widespread. Second, personal preventive measures are recommended to prevent community transmission. Third, serial viral testing is recommended in long-term care facilities to identify cases, isolate positive cases, quarantine contacts, and thus prevent outbreaks. Overall, adherence to these measures can reduce the risk of SARS-CoV-2 transmission [13-17].

### SARS-CoV-2 Vaccines

Vaccines have been crucial tools in the prevention of viral infections for a long time. The development of vaccines to prevent SARS-CoV-2 infection is seen as a highly promising strategy for mitigating the COVID-19 pandemic. By the end of 2020, multiple vaccines had become available for use, and over 40 candidate vaccines were being tested in human trials, with more than 150 in preclinical trials [18]. Several platforms have been used to develop COVID-19 vaccines through traditional methods, such as inactivated or live attenuated viruses, as well as modern platforms, such as recombinant proteins and vectors [19].

### COVID-19 Vaccine Availability and Delivery in Africa

According to the US Food and Drug Administration (FDA) and the WHO, to be licensed and approved for use, a COVID-19 vaccine must meet a minimum efficacy criterion of at least 50%, with a lower bound of the 95% CI at 30% [20,21]. To determine efficacy, phase 3 trials are being conducted with a follow-up period of 6 months, and a minimum of 30,000 participants are needed. Once phase III trials demonstrate safety and efficacy according to the minimum efficacy criteria, the FDA makes decisions on vaccine licensure. Regulatory bodies in Canada and European countries also take similar approaches to licensing their vaccines [20]. However, whether Africa can meet these licensing requirements remains unknown. Vaccine manufacturing requires sophisticated technological resources. Although the production of an African-made COVID-19 vaccine is encouraged to address the pandemic effectively, many countries’ vaccine manufacturing capabilities need to be improved with government funding [22-24], which is meagerly the case among most African governments. The storage and handling requirements of vaccines pose operational challenges, particularly in resource-limited settings, such as Tanzania. For example, the Pfizer vaccine requires ultracold storage in specialized freezers. Additionally, the WHO recommends discarding open vials 6 hours after opening or at the end of the immunization session, whichever comes first. The need for vials used to hold vaccines may also pose supply chain issues in Africa. This highlights the need for more African-made vaccines that can consider storage and handling challenges, in addition to other economic, logistical, and sociocultural issues [25]. Owing to limited vaccine supplies, recipient groups in the beginning were prioritized based on the risks of acquiring infection, severe morbidity and mortality, negative societal impact, and transmission to others. The WHO has proposed a framework that takes into account global equity concerns, including the assurance of vaccine access to low- and middle-income countries [21].

The development of vaccines within African countries could help increase their availability and accessibility in the region. One of the initiatives in place is the development of the TANCoV-1.3.20 vaccine candidate in Tanzania.

### The TANCoV-1.3.20 Vaccine

The TANCoV-1.3.20 is a live attenuated vaccine derived from a novel strain of avian coronavirus (ACoV). The virus was isolated in 2020 in Tanzania from chickens identified with ACoV disease, and confirmed by polymerase chain reaction using spike protein–encoding gene (S1) primers, which revealed a band of 213 base pairs. Preliminary results have shown that the strain TANCoV-1.3.20 cross-reacted with and was neutralized by antibodies from individuals who had recovered from SARS-CoV-2 infection. Results from the virus neutralization test revealed that the geometric mean titer (GMT) of neutralizing antibodies detected in individuals recovered from SARS-CoV-2 infection was log_2_ 6.5.

It has been shown that protective immunity provoked by ACoV is associated with the major structural protein, spike (S) glycoprotein, which induces neutralizing antibodies [26]. Neutralizing antibodies against the S-protein have been targeted for the development of protective vaccines and therapies against SARS-CoV-2 infection worldwide.

It has been proposed that the ACoV vaccine may be used against SARS-CoV-1 [27]. Moreover, ACoV does not cause disease in humans [28].

The TANCoV 1.3.20 vaccine is thermostable, can be stored at ambient temperatures, and can be administered via the intranasal route while maintaining safety and stability. Owing to its route of administration, there is no need to use needles or syringes, thereby avoiding needle-stick injuries and disposal challenges. It is in liquid form with a light green color. The dose of the vaccine per nasal drop (25 µL) has a titer of 10^6^ embryo infective dose (EID_50_) mL^–1^.

On the basis of preclinical results from animal models of inbred mice, rabbits, and pigs, the TANCoV-1.3.20 vaccine is tolerable, safe, and immunogenic.

## Methods

### Study Design and Settings

This is a phase 1/2a double-blinded, randomized controlled phase trial that aims to assess the safety, tolerability, and immunogenicity of the TANCoV-1.3.20 SARS-CoV-2 vaccine candidate among healthy participants in the Dar es Salaam and Mbeya regions of Tanzania. In Dar es Salaam, participants are recruited from the general population and communities such as the police and prison forces, while in Mbeya, participants are recruited from the general population.

For the social component, a convergent mixed methods study using separate and parallel administrations of semistructured in-depth interviews and interviewer-administered questionnaires is embedded in the trial. The design enables the generation of an in-depth understanding of aspects of the trial that promote participants’ acceptance of trial participation. The theoretical framework of acceptability (TFA) will be used to guide the assessment of acceptability. The TFA consists of 7 component constructs, namely, affective attitude, burden, ethicality, intervention coherence, opportunity costs, perceived effectiveness, and self-efficacy [29].

A list of all enrolled participants per site, including their demographics and scheduled visits, will be developed to aid in purposive sampling and the scheduling of appointments for informed consent and data collection during follow-up visits.

### Study Objectives and End Points

#### Objectives

The objectives of the study are (1) to determine the safety of TANCoV-1.3.20 at a dose of 100 µL with or without a booster and 200 µL without a booster delivered nasally, (2) to assess the tolerability of TANCoV-1.3.20 at a dose of 100 µL with or without a booster and 200 µL without a booster delivered nasally, (3) to determine the immunogenicity of TANCoV-1.3.20 at a dose of 100 µL with or without a booster and 200 µL without a booster delivered nasally, (4) to compare the immunogenicity of TANCoV-1.3.20 at a dose of 100 µL with or without a booster and 200 µL without a booster delivered nasally, (5) to explore the factors influencing the willingness and acceptability of participants to participate in local COVID-19 vaccine trials, and (6) to assess the perceptions of study participants and their social environment toward the TANCoV-1.3.20 vaccine.

#### Primary End Points

Safety will be assessed using clinical and laboratory parameters. We will grade clinical, hematological, and biochemical tests, as well as evaluate the frequency, severity, and duration of adverse events (AEs). AEs will be graded according to the Division of AIDS toxicity grading table, version 2.1, July 2017.

##### Safety End Points: Incidence of AEs (Safety and Tolerability)

The safety end points are (1) the proportion of participants with local and systemic solicited AEs from days 1 to 7 after each intraperitoneal (IP) administration; (2) the proportion of participants with grade 2 or higher unsolicited AEs, including safety laboratory (biochemical and hematological) parameters, from the day of each IP administration through 28 days after IP administration; and (3) the proportion of participants with serious AEs (SAEs) throughout the study period.

##### Secondary End Points: Immunogenicity End Points

The secondary end points are (1) the proportion of participants with a specific TANCoV-1.3.20 humoral response, (2) the magnitude of the specific cellular response to TANCoV-1.3.20, (3) the proportion of participants with serum-neutralizing antibody responses to TANCoV-1.3.20, and (4) the characteristics of the humoral response produced by the TANCoV-1.3.20 vaccine.

##### Capacity Building and Social Aspects of the Trial End Points

The capacity building and social aspects of the trial end points are (1) the number of personnel who acquired international critical skills, knowledge, and strengthened infrastructure for conducting a clinical trial; and (2) the perception, willingness, and acceptability of the TANCoV-1.3.20 vaccine trial among study participants and their social environment.

### Sample Size Estimation

The sample size for this noninferiority clinical trial was calculated to compare the proportion of seroconversion on day 28 between participants receiving the investigational vaccine TANCoV-1.3.20 and those receiving the standard Sinopharm COVID-19 vaccine. On the basis of prior studies and seroconversion data from the Tanzanian setting, we assumed an 88% seroconversion rate for the control group (Sinopharm) and expected a 90% seroconversion rate in the experimental group. A noninferiority margin of 20 percentage points was set, meaning that TANCoV-1.3.20 would be considered noninferior if its seroconversion rate was not more than 20% lower than that of Sinopharm. Using a 1-sided significance level (α) of .05, 80% power (1−β), and a 1:1 allocation ratio (experimental: control), the required total sample size was estimated at 50 participants per group. The study includes 3 study groups, with 25 participants per arm, resulting in a total planned sample size of 150 participants. This sample size provides sufficient power to establish noninferiority if the lower bound of the CI for the difference in seroconversion remains above the preset margin. The calculation assumes binomial proportions and follows standard methods for noninferiority designs in parallel trials [30].

### Study Groups

Table 1 below outlines the allocation of participants across the 3 study groups, comparing intranasal TANCoV and intramuscular Sinopharm vaccines. Each group consisted of 2 arms with a 3:1 randomization to favor the experimental TANCoV vaccine, totaling 150 participants.

**Table 1.** Participant study groups and investigational product dosages for each group.

Group and arm	Sample size, n	Vaccination (day 0)	Booster (day 28)
1			
A (control)	25	Sinopharm (IM^a^)+100 µL saline (nasal)	Sinopharm (IM)
B (TANCoV-1.3.20)	25	TANCoV-1.3.20) 100 µL (nasal)+saline (IM)	TANCoV-1.3.20) 100 µL (nasal)
2			
A (control)	25	Sinopharm (IM)+100 µL saline (nasal)	Sinopharm (IM)
B (TANCoV-1.3.20))	25	TANCoV-1.3.20) 100 µL (nasal)+saline (IM)	Saline (IM)
3			
A (control)	25	Sinopharm (IM)+200 µL saline (nasal)	Sinopharm (IM)
B (TANCoV-1.3.20))	25	TANCoV-1.3.20) 200 µL (nasal)+saline (IM)	Saline (IM)

a IM: intramuscular.

With any addition of study participants, an equal number of participants will be randomly included in each of the 3 arms. Participant replacement will be considered in the following circumstances to allow primary analysis to continue as planned: (1) early study termination, (2) loss to follow-up during study screening and the enrollment period, and (3) the loss of samples (blood, peripheral blood mononuclear cells [PBMCs], serum, and nasal swabs) or temperature during transportation from the study site to the central laboratory, which renders them unavailable for analysis. In the course of enrollment and vaccination, sample quality necessitated the increase of sample size to 169; the additional samples were randomly included in each group.

### Randomization

Eligible participants will be randomized to the intervention arm or control arm via permuted block randomization with a block size of 6 stratified by site. This method ensures a balanced allocation of participants among the groups. Participants fulfilling the eligibility criteria will be randomized sequentially, and each participant will have a unique randomization code. A randomization schedule will be created by an independent statistician via permuted-block randomization of variable size.

Before enrollment, the study screening electronic case report form must be completed. When a participant is confirmed to be eligible and has consented to be enrolled, their details will be entered on the next available line of the enrollment register to ascertain the trial number assigned to that participant, which will be used on all trial documents. This number is unique and will not be used for any other participant.

### Blinding

Blinding will be performed for the investigators and participants. Participants receiving the IP through nasal drops will also be provided with a normal saline injection, which is intended to prevent unblinding. Those who receive the Sinopharm vaccine through intramuscular injection will receive normal saline nasal drops (placebo nasal drops), which are also intended to prevent unblinding. Unblinding will be discouraged during vaccination; however, if a trial clinician considers it necessary for a participant’s allocated vaccination to be unblinded, this should first be discussed with the principal investigators and other key investigators. If unblinding is deemed appropriate, the reason for doing so should be recorded. In the event that adverse vaccine reactions are considered possibly related to the study vaccine, the study vaccine will be discontinued for the affected participant.

### Study Sites

The clinical sites will be located at Mwananyamala Regional Referral Hospital in Dar es Salaam for the National Institute of Medical Research (NIMR) Muhimbili and the NIMR-Mbeya Medical Research Center in Mbeya, Tanzania.

For the Dar es Salaam site, all safety laboratory tests will be performed at the Muhimbili National Hospital Central Pathology Laboratory, and PBMC processing will be conducted at the National Public Health Laboratory. For the Mbeya site, laboratory tests will be carried out at the NIMR-Mbeya Medical Research Center safety laboratory. All samples for immunogenicity analysis will be analyzed at the NIMR-Mbeya Medical Research Center Immunology Laboratory.

### Inclusion and Exclusion Criteria

Inclusion criteria are (1) healthy individuals aged 18 to 45 years at screening and who are willing to provide written informed consent, (2) individuals whose BMI falls between 18.0 and 34.9 kg/m^2^, (3) willingness to undergo COVID-19 testing and negative antigen and antibody tests, (4) satisfactory completion of an assessment of understanding before enrollment, (5) resident in Dar es Salaam or Mbeya and willing to remain in the same region for the duration of the study, and (6) consistent use of effective contraception for individuals who can become pregnant and should have a negative pregnancy test at enrollment.

Exclusion criteria are (1) active tuberculosis or other systemic infectious processes elicited by a review of systems, physical examination, and laboratory testing; (2) history of immunodeficiency or chronic illness requiring continuous or frequent medical intervention; (3) HIV, hepatitis B, and/or hepatitis C infection; (4) received blood or blood products or immunoglobulin in the past 3 months; (5) hives or recurrent hives and severe eczema; (6) history of psychiatric, medical, and/or substance abuse problems during the past 6 months; (7) history of grand mal epilepsy or currently taking antiepileptic medications; (8) receiving immunosuppressive therapy; (9) have received experimental therapeutic agents, currently or within the last few months; (10) previously received COVID-19 candidate vaccines such as Janssen, Sputnik, SINOVAC, Sinopharm, Pfizer, and Moderna, or received any live attenuated vaccine within 90 days before enrollment; (11) history of severe local or general reactions to vaccination; (12) lactating mothers; and (13) individuals deemed unlikely to comply with protocol procedures as judged by the investigators.

### Study Procedures

#### Overview

Briefing sessions containing general information about the study, counseling, and prescreening procedures will be conducted at the trial site clinic and different venues in the community, including open spaces and police or prison facilities. All trial-related activities and vaccinations will take place at the clinical trial sites. Before the study procedures, participants will go through the consent process and will sign 2 copies of the informed consent form. One copy will be kept at the trial site, and the other copy will be provided to the study participant. TANCoV-1.3.20 vaccinations will be administered 4 weeks apart for individuals who will receive the booster dose, and the total follow-up duration will be 6 months. No further vaccinations will be administered in cases of consent withdrawal, confirmed pregnancy, or adverse drug reactions considered possibly related to the study vaccine.

#### Sample Collection

Blood, urine, saliva, and nasopharyngeal samples will be collected according to the visit schedule. These samples will be collected as per specific trial standard operating procedures.

#### Pregnancy Reporting and Follow-Up

Female participants will be required to use an effective contraceptive method starting 1 week before the first vaccination and continuing until 3 months after the last vaccination. A pregnancy test will be performed according to the study flow schedule. For participants who become pregnant before completing the vaccine series, no further vaccinations will be administered. A positive urine pregnancy test result will be communicated to the participant and the sponsor. The participant will be monitored for all the remaining scheduled visits according to the schedule of procedures for safety evaluation, as well as for maternal outcomes during pregnancy, labor, delivery, and postdelivery periods. The infant’s outcome will also be documented on the pregnancy outcome form.

#### Safety Assessment and Reporting

Safety assessment will be based on monitoring AEs, complications, and side effects, which will be collected throughout the study. Any AEs or SAEs encountered will be reported and categorized by the reviewing ethics committee and the Division of AIDS toxicity table for grading the severity of adult and pediatric adverse events.

Local and systemic assessments will be recorded starting 30 minutes after vaccination at the following intervals: 30 minutes as direct observation after vaccination at the clinic and days 1 to 7 at the clinic or home via the reactogenicity form and diary cards at the clinic and home, respectively. All other medical events, including laboratory safety assessments throughout the entire study period, will be reported on the AE form.

#### Immunogenicity Assessment

The processed samples (sera) will be analyzed to evaluate immunoglobulin G (IgG) and immunoglobulin M (IgM) titers using the WANTAI SARS-CoV-2 Ab enzyme-linked immunosorbent assay. The resulting antibody concentrations will be quantified, and the values will be expressed as GMTs to provide a reliable summary measure of the immune response while minimizing the effect of extreme values or outliers.

In addition to humoral responses, the cell-mediated immune response will be characterized to provide insights into T-cell activation and cytokine production following vaccination. This will include an interferon-γ (IFN-γ) enzyme-linked immunospot assay to quantify antigen-specific T-cell responses, alongside an intracellular cytokine staining assay performed on cryopreserved PBMCs. The intracellular cytokine staining assay allows detailed profiling of cytokines such as IFN-γ, interleukin-2 (IL-2), tumor necrosis factor-α (TNF-α), and other relevant markers, enabling the evaluation of T-cell subsets (cluster of differentiation 4 [CD4^+^] and cluster of differentiation 8 [CD8^+^]) involved in vaccine-induced immunity.

All immunology work and long-term sample storage, for at least 15 years, will be centralized at the NIMR-Mbeya Center.

### Data Management

#### Overall Data Management

All the data will be collected and managed electronically via secure REDCap (Research Electronic Data Capture; Vanderbilt University) software. The data will be handled confidentially, and no names or any other identifying information will be used during report writing, results dissemination, or publication.

The standard operating procedures will be followed in all laboratories to ensure the quality of the data. All results from the laboratory will be reviewed and signed by the laboratory manager for completeness and correctness before being entered into the electronic system. Clinical laboratory results will be entered into an electronic database by the clinician after review.

The location of the data management center is Dar es Salaam at the NIMR Muhimbili Center. Data from both sites will be centrally checked for inconsistencies and logical errors by site supervisors before being uploaded. Copies, including documentation detailing all the queries and changes, will be stored in a study-specific binder at the trial coordination site. The data generated from the mixed methods component will be managed at NIMR-Mbeya, where audio-recordings and transcripts will be kept in password-protected folders within the center’s server.

#### Data Analysis

The data will be analyzed using Stata (version 14; StataCorp LLC). Findings will be reported according to the CONSORT (Consolidated Standards of Reporting Trials) guidelines. Primary analyses will be based on the intention-to-treat population, and secondary analyses will be based on the per-protocol population.

The primary end point will be analyzed via a marginal Poisson regression model fitted using generalized estimating equations, with the logarithm of follow-up time included as an offset and the study arm as the predictor. The incidence rate ratio and its 95% CI will be derived from the Poisson regression model. Incidence rates will also be calculated as the number of incident cases divided by the person-time in years, with person-time calculated from randomization to (1) the study end at 6 months after the last participant enrollment and (2) death or the time last seen if lost to follow-up. In addition, absolute effects on the primary endpoint will be summarized using risk differences (events per 1000 person-years) and their 95% CIs, estimated from the model-based predicted rates in each arm.

In a secondary analysis of the primary end point, we will adjust the Poisson regression model for covariates, defined a priori (age and sex). Each protocol analysis will be performed and defined according to reported adherence to the intended allocation. Subgroup analysis of the primary end point will also be performed via the Poisson model on the prespecified subgroup variables (age and sex).

Other secondary outcomes will be analyzed using generalized linear models, depending on their distributions. The mean difference with its 95% CI will be derived for a continuous outcome, and the odds ratio with its 95% CI will be estimated for a binary outcome.

AEs will be summarized according to the number and percentage of individuals experiencing AEs.

The quantitative and qualitative data for the mixed methods component will be analyzed separately and integrated for interpretation. The interviewer-administered questionnaires will be entered into REDCap and analyzed using Stata, mainly through descriptive analysis. Demographic characteristics will be summarized via descriptive statistical methods, where continuous data will be summarized by the mean, SD, median, and IQR, and categorical values will be summarized by frequency and percentages. A mean score of general acceptability will be computed; however, each of the responses to the 7 constructs of the TFA will be analyzed based on the proportions of acceptance and nonacceptance.

Thematic analysis will be adopted as an analytic technique for qualitative data with the use of ATLAS software (version 8.4.26; ATLAS.ti Scientific Software Development GmbH). The analysis will also be guided by the TFA. This entails verbatim transcription of audio recordings and translation into English, as well as coding of transcripts where emergent and preconceived codes, on the basis of the TFA, are attached to the transcripts. Thereafter, a first draft of the codebook, containing a list of codes and their explanations, will be developed prior to coding based on the TFA constructs. Thereafter, the first 2 transcripts of participants from each of the 2 sites were coded independently by 4 social scientists (2 from each site). Discrepancies in coding will be resolved through a discussion among social scientists, and an agreement on the coding will be sought to further refine the codebook. Emergent codes arising from the transcripts will also be added to the codebook.

Upon completion of coding, codes are grouped into subcategories and categories on the basis of similarities that exist among a group of codes in terms of content or context. This process involves the development of subthemes and themes that reflect the underlying meanings attributed to the categories. Upon completion of separate analyses of quantitative and qualitative data, the results of each dataset are integrated using a weaving approach.

#### Statistical Considerations and Analysis

##### Statistical Analysis

An interim review of the data will be performed by the independent data and safety monitoring committee. This will be done when at least 80% of the participants have been followed for at least the first 3 months. This is beyond the halfway stage to ensure that the safety estimate calculated at this time is statistically stable. Early stopping is considered if the significance level is *P*=.001 or lower (ie, if the Haybittle-Peto boundary is used).

The final analysis will be conducted once all study participants have received all their vaccinations, completed the final visit, or permanently withdrawn from the study. Analyses will be based on the principles of intention-to-treat and per protocol.

Baseline characteristics will be summarized by study groups using appropriate summary statistics. The study participant characteristics to be summarized will include demographic variables and baseline values relevant to safety and immunogenicity. The number and percentage of participants experiencing any AEs or SAEs will be summarized by severity and their relationship with the study treatment for each group. The calculation of incidence rates of AEs will include each participant only once, either according to the worst severity or the first reported event. Rates of the various AEs will be compared between the study groups via the Fisher exact test and will be summarized via exact binomial CIs. Safety, hematological, and clinical chemistry laboratory parameters will be summarized at the study visit according to the study group. Comparisons will be performed using 2-sample *t* tests or nonparametric equivalents as appropriate for the data.

##### Immunogenicity Analysis

The primary endpoints of the immunogenicity analysis will focus on quantifying and comparing immune responses elicited by the vaccine across study groups and time points. The seroconversion rate will be determined as the proportion of participants who achieved a predefined antibody threshold postvaccination relative to their baseline levels, indicating the vaccine’s ability to induce a measurable immune response. In addition, GMTs for antibody concentrations or the mean fluorescence intensity for flow cytometry–based assays will be calculated to summarize central tendencies of immune responses while minimizing the influence of extreme values. These parameters will be statistically compared across treatment arms, dose groups, and time points via appropriate statistical tests such as paired 2-tailed *t* tests, Wilcoxon signed-rank tests, or one-way ANOVA, depending on the data distribution. Additionally, to assess immune kinetics, longitudinal models, which describe patterns of immune waning or boosting following primary and subsequent vaccine doses, will be used. Furthermore, correlation analyses, including protection against infection during the entire study period, will be performed to explore the relationship between antibody levels and clinical outcomes, thereby identifying potential immunological correlates of protection and revealing the predictive value of measured immune parameters.

### Study Monitoring

There will be internal and external monitoring activities as determined by the trial management group. Monitoring activities will include a review of the study progress, AEs, reactogenicity, protocol deviations, laboratory data, and other data available in the database. In addition, visits will include reviewing essential study documents such as participant binders, source documents, regulatory files, case report forms, and study clinics and pharmacies.

### Protocol Deviations and Violations

All protocol deviations and violations encountered during the study will be documented and reported to the sponsor, the ethics review committee, and regulatory authorities.

### Study Completion

At the end of the study, a summary of the study results will be sent to the institutional review board or institutional ethics committee, along with all the required reports, as appropriate, and all the reports will be sent to the regulatory authorities, as applicable. After closing or completing the study, all documents, intervention allowances, and decoding of information, summaries, and reports for the institutional review board or institutional ethics committee and regulatory authorities will be retained in the investigator site file.

The SPIRIT (Standard Protocol Items: Recommendations for Interventional Trials) reporting guidelines were used in preparing this study (Checklist 1) [31].

### Ethical Considerations

All participant information will be handled confidentially, and no information that identifies the participant will be revealed to the sponsor or unauthorized personnel. Individual participants will not be identified in the resulting publications and presentations from the trial. The trial will comply with the principles of the Data Protection Act of the country.

This protocol has received ethical approval from the National Health Research Ethics Committee at NIMR (NIMR/HQ/R.8a/Vol. IX/381 and NIMR/HQ.8b/Vol. I/1084), the Mbeya Medical Research and Ethics Committee (SZEC-2439/R.C/V.1/136 and SZEC-2439/R.C/V.1/66), and the Tanzania Medicines and Medical Devices Authority (TMDA-WEB0021/CTR/0012/03 and BC.69/96/21/01). Informed consent will be obtained from the participants as per the protocol.

Regular compensation will be provided to participants to cover their travel, inconveniences, and lunch expenses. For scheduled and unscheduled visits, this amounts to 30,000 Tanzanian shillings (TShs; US $1=2421 TShs) for Dar es Salaam participants and 25,000 TShs for Mbeya participants. If any medication is required as a result of the study, it will also be provided free of charge. In case of hospitalization or if further care is needed, these patients will be covered under the National Insurance Corporation of Tanzania Limited and will be confined to the period of the study.

## Results

### Trial Status

This study describes the protocol for a TANCoV-1 phase 1/2a double-blinded, randomized controlled trial conducted in the Dar es Salaam and Mbeya regions of Tanzania. The trial is designed to enroll 150 healthy participants aged 18 to 45 years and randomize them in a 1:1 ratio into 3 groups to receive TANCoV-1.3.20 at a dose of 100 µL with a booster, 100 µL without a booster, or 200 µL without a booster. Follow-up is planned for 6 months postvaccination, and analyses will be conducted in Stata in accordance with CONSORT reporting standards.

### Recruitment and Study Progress

As of February 2024, recruitment has been completed (N=169). Laboratory testing is ongoing, and data cleaning and statistical analyses are underway. Safety and immunogenicity outcomes will be analyzed after all follow-up visits and laboratory assays have been completed.

### Anticipated Reporting

Final analyses are expected to be completed by December 2026, and the primary results are expected to be submitted for publication in 2025. Study findings will be reported following CONSORT guidelines, including results for the primary end points (incidence of AEs and immunogenicity outcomes reflecting local, humoral, and cellular immune responses).

## Discussion

The COVID-19 pandemic has had a profound impact globally, causing enormous morbidity and mortality while posing unprecedented challenges to economic growth [3]. Rapid vaccine development and an emphasis on mass vaccination have been promising strategies for reducing the severity of the disease as well as minimizing the transmission of the virus. However, there have been some setbacks in the process of vaccination, including, among others, vaccine storage and handling challenges, particularly in low-income countries [32]. In addressing the current gaps and disparities in COVID-19 vaccines among the Global North and South, and as part of preparedness for future pandemics that might require locally developed solutions, several African governments have made efforts in the research and development of locally made solutions, including vaccines. One such endeavor is the production of Tanzania-made COVID-19 vaccines, and TANCoV-1.3.20 is a SARS-CoV-2 vaccine.

As part of the effort to address these setbacks, this clinical trial intends to assess the safety, tolerability, and immunogenicity of TANCoV-1.3.20, a SARS-CoV-2 vaccine, in Tanzania. To assess the potential impact of the vaccine, local, humoral, and cellular immune responses will be measured. Notably, this clinical trial will use TANCoV-1.3.20, a SARS-CoV-2 vaccine that can be administered intranasally, which is a noninvasive mode of administration. Methodologically, in a double-blind manner, some participants will receive the IP intranasally, while the control group will be administered Sinopharm as a standard of care through intramuscular injection. For blinding purposes, normal saline will be given both intranasally and via injection. Methodologically, we anticipate that participants might be hesitant to receive an injection, mainly due to fear associated with the injection and foreign vaccine hesitancy due to myths associated with COVID-19 vaccines. The trial team has a plan to mitigate this by providing education and addressing the fear of needle pricks. In addition, as COVID-19 persists, we anticipate challenges in obtaining COVID-19-negative participants; therefore, a significant number of individuals will need to be screened to identify COVID-19-negative participants.

Evaluating this vaccine will provide valuable data as a step toward ensuring that cost-effective vaccines with easy storage and handling requirements, yet effective against COVID-19, are available to all resource-limited areas, as is the case in most African countries. The findings of this trial will help increase public acceptance of the COVID-19 vaccine, thereby contributing to the ongoing global effort to combat the COVID-19 pandemic.

## Supplementary material

10.2196/81323 Checklist 1 SPIRIT checklist.

## Abbreviations

ACoV: avian coronavirus
AE: adverse event
CD4^+^: cluster of differentiation 4
CD8^+^: cluster of differentiation 8
CONSORT: Consolidated Standards of Reporting Trials
FDA: Food and Drug Administration
GMT: geometric mean titer
IFN-γ: interferon–gamma
IgG: immunoglobulin G
IgM: immunoglobulin M
IL-2: interleukin-2
IP: intraperitoneal
MERS-CoV: Middle East respiratory syndrome coronavirus
NIMR: National Institute of Medical Research
PBMC: peripheral blood mononuclear cell
REDCap: Research Electronic Data Capture
SAE: serious adverse event
SPIRIT: Standard Protocol Items: Recommendations for Interventional Trials
TFA: theoretical framework of acceptability
TNF-α: tumor necrosis factor–alpha
WHO: World Health Organization
